# Dissipation of excess excitation energy of the needle leaves in *Pinus* trees during cold winters

**DOI:** 10.1007/s00484-016-1182-3

**Published:** 2016-05-19

**Authors:** AO Zhang, Zhen-Hai Cui, Jia-Lin Yu, Zi-Ling Hu, Rui Ding, Da-Ming Ren, Li-Jun Zhang

**Affiliations:** 1 College of Biological Sciences and Technology, Shenyang Agricultural University, Shenyang, 110866 China; 2 College of Agronomy, Shenyang Agricultural University, Shenyang, 110866 China; 3 Liaoning Province Research Center of Plant Genetic Engineering Technology, Shenyang, 110866 China

**Keywords:** *Pinus koraiensis*, *Pinus tabulaeformis*, *Pinus armandi*, Chlorophyll fluorescence, Photoinhibition, Absorption spectrum, Xanthophyll cycle, Photosystem complex

## Abstract

Photooxidative damage to the needle leaves of evergreen trees results from the absorption of excess excitation energy. Efficient dissipation of this energy is essential to prevent photodamage. In this study, we determined the fluorescence transients, absorption spectra, chlorophyll contents, chlorophyll *a*/*b* ratios, and relative membrane permeabilities of needle leaves of *Pinus koraiensis*, *Pinus tabulaeformis*, and *Pinus armandi* in both cold winter and summer. We observed a dramatic decrease in the maximum fluorescence (*F*_m_) and substantial absorption of light energy in winter leaves of all three species. The *F*_m_ decline was not correlated with a decrease in light absorption or with changes in chlorophyll content and chlorophyll *a*/*b* ratio. The results suggested that the winter leaves dissipated a large amount of excess energy as heat. Because the cold winter leaves had lost normal physiological function, the heat dissipation depended solely on changes in the photosystem II supercomplex rather than the xanthophyll cycle. These findings imply that more attention should be paid to heat dissipation via changes in the photosystem complex structure during the growing season.

## Introduction

Evergreen pine (*Pinus* spp.) trees are widely distributed throughout the world and play important ecological roles. Although some species have stronger tolerance to cold than others, their needle leaves are often damaged during winter (Öquist and Huner 1991; Lehner and Lütz 2003; Rammig et al. 2010). This damage is due not only to low temperature, but also to strong light (García-Plazaola et al. 1999; Blennow and Lindkvist 2000), with intense radiation often compounding the damage by low temperature (Yamazaki et al. 2003). In contrast, the needle leaves can endure lower temperatures under weak illumination. Light is the energy source for plant photosynthesis, but light energy will damage the photosynthetic apparatus when more is absorbed by leaves than is consumed by photosynthesis (Murata et al. 2007). This process is referred to as photoinhibition. The excess excitation energy that is not used by photosynthesis generates reactive oxygen species (ROSs) and results in photooxidation damage of the photosynthetic apparatus (Nishiyama et al. 2001; Horton 2012; Tyystjarvi 2013). The degree of the photodamage depends on the clearance of ROSs (Mishra et al. 1993; Hwang et al. 2004; Murata et al. 2012), protection of the photosynthetic apparatus (Murchie and Niyogi 2011; Ruban et al. 2012), and repair of damaged structures and proteins (Takahashi and Badger 2011; Allahverdiyeva and Aro 2012; Goh et al. 2012). However, the first line of defense against photodamage is to decrease the excess energy.

Plants have several mechanisms to avoid excitation energy surplus, including: (1) decreasing the absorption of light energy (Jiang et al. 2006) by, in some species, altering the angle of their leaves under strong light (Rubio et al. 2007); (2) enhancing photorespiration (Osmond et al. 1997); and (3) increasing the dissipation of excess energy (Allahverdiyeva and Aro 2012). In the growing season, some plants can engage all three mechanisms. However, in cold winters with very low temperature (−10 to −20 °C), evergreen leaves lost or seriously weaken the ability to regulate their physiological activities and depend mainly on the dissipation to reduce the energy surplus.

The excess energy absorbed by plant leaves is dissipated as fluorescence and heat (Allahverdiyeva and Aro 2012). Fluorescence release is common when photosynthesis is impeded by strong light. The amount of released fluorescence varies with the changes in the structure of the photosystem complex, for instance, the disassociation of the photosystem II (PSII) light-harvesting complex from the PSII supercomplex (Tikkanen and Aro 2012). For heat dissipation during the growth season, the excess energy is released via the xanthophyll cycle (Eskling et al. 1997; Jahns and Holzwarth 2012), which involves the enzymatic interconversion between violaxanthin and zeaxanthin in higher plants and depends on the pH differential across the thylakoid membrane (Bratt et al. 1995; Büch et al. 1995). However, the same leaves of evergreen trees are quite different in physiological status such as photosynthesis, respiration, enzyme activity, and membrane permeability between in cold winter and in summer. Therefore, they may have a different mechanism of excess energy dissipation and the process in the cold winter might involve less enzymatic reactions. In this experiment, we compared the fluorescence transients, light absorption spectra, chlorophyll contents, and relative membrane permeabilities of the needle leaves of three *Pinus* species in the cold winter and summer to analyze their mechanisms of excess energy dissipation during cold winter.

## Materials and methods

### Plant samples

The plants used in this study were specimens of *Pinus koraiensis* (height 7.2–8.5 m, diameter at breast height 16.3–18.1 cm, tree age 48 years), *Pinus tabulaeformis* (height 10.1–11.3 m, diameter at breast height 26.9–29.4 cm, tree age 31 years), and *Pinus armandi* (height 7.9–9.1 m, diameter at breast height 21.0–23.3 cm, tree age 41 years) growing in the botanic garden of Shenyang Agricultural University, Shenyang city, Liaoning Province, China (41° 82′ N, 123° 56′ E). *P. koraiensis* and *P. tabulaeformis* are native species and widespread in the northeast of China (38°N-56°N) and *P. armandi* is an introduced species with less cold resistance. Three plants were chosen for measurement from each species. Twigs were cut off from the top of branches on the sunlit side and taken to the laboratory. The test was replicated three times. The winter samples were taken in late January 2012, when the air temperature ranged from −20 to −7 °C (average, −17.8 °C). The summer sampling was in late June 2013 (air temperature, 18–31 °C; average 23 °C).

### Measurement of fluorescence transients

Needle leaves from the bases of twigs were subjected to darkness for 15 min and then exposed to 3000 μmol m^−2^ s^−1^ photon flux density generated by a Plant Efficiency Analyzer (Handy-PEA) (Hansatech, Kings Lynn, Norfolk, UK) for 1 s (Strasser and Strasser 1995) to determine fluorescence induction curves. According to the fluorescence transients (OJIP) (Guissé et al. 1995; Strasser et al. 2004), the following parameters were obtained: (1) *F*_o_, the initial fluorescence yield; (2) *F*_m_, the maximum fluorescence; and (3) *F*_v_, the variable fluorescence.

### Measurement of absorbance spectra, chlorophyll content, and relative membrane permeability

Absorbance spectra were measured with a portable Unispec SC spectrometer (PP Systems, Amesbury, MA, USA). Data were analyzed by Multispec 5.1.5. Chlorophyll content and the chlorophyll *a*/*b* ratio were measured according to Arnon (1949). The relative membrane permeability of the middle parts of needle leaves was measured according to Bao et al. (2009).

### Data analysis

Statistical analyses were performed using SPSS 13.0 (IBM, Chicago, IL, USA).

## Results

### Fluorescence transients and parameters

Fluorescence transients reflect the excitation energy distribution in the photosynthetic energy absorption and transfer system. As shown in Fig. 1, the needle leaves in cold winter showed atypical OJIP curves. The values of the O points (*F*_o_), P points (*F*_m_) and *F*_v_ (*F*_m_ − *F*_o_), and the ranges of the *F*_v_/*F*_m_ ratio in the winter needles were much lower than in the summer ones (Table 1). The analysis of variance showed that the differences of these chlorophyll fluorescence parameters were significant or extremely significant between in the winter and summer as shown in Table 1. These results suggested that the photochemical activities (*F*_v_) in the winter needles were extremely weak. Notably, *F*_m_ values in the winter were remarkably lower than in the summer; this decrease might result from lower light energy absorption or greater release of absorbed energy release in forms other than fluorescence.

**Fig, 1:**
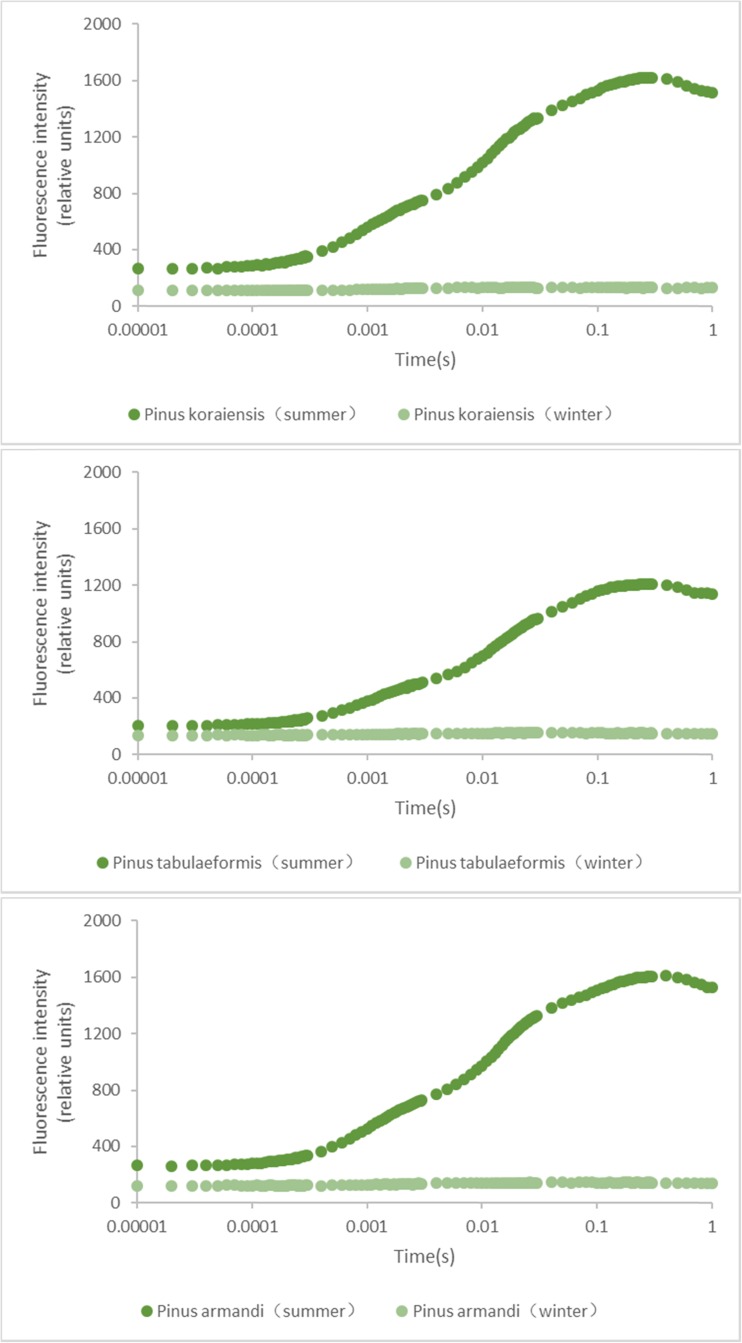
The comparison of chlorophyll fluorescence transients of needles in *Pinus* trees between in cold winter and summer

**Table 1:** The comparison of chlorophyll fluorescence parameters of needles in *Pinus* trees between in cold winter and summer

Species	Season	Fo	Fm	Fv	Fv/Fm
*P. koraiensis*	Winter	109 ± 19.85**	136 ± 25.02**	27 ± 10.30**	0.20 ± 0.06**
	Summer	260 ± 27.83**	1621 ± 203.44**	1361 ± 180.10**	0.84 ± 0.01**
*P. tabulaeformis*	Winter	135 ± 22.50**	154 ± 17.78**	18.67 ± 4.73**	0.12 ± 0.05**
	Summer	200 ± 15.93**	1209 ± 83.78**	1009 ± 76.01**	0.83 ± 0.01**
*P. armandi*	Winter	121 ± 3.06**	147 ± 6.93**	26.33 ± 4.16**	0.17 ± 0.02**
	Summer	255 ± 27.91**	1625 ± 213.53**	1370 ± 215.53**	0.84 ± 0.03**

**Significance at *P* ≤ 0.01

### Absorption spectra

**Fig. 2:**
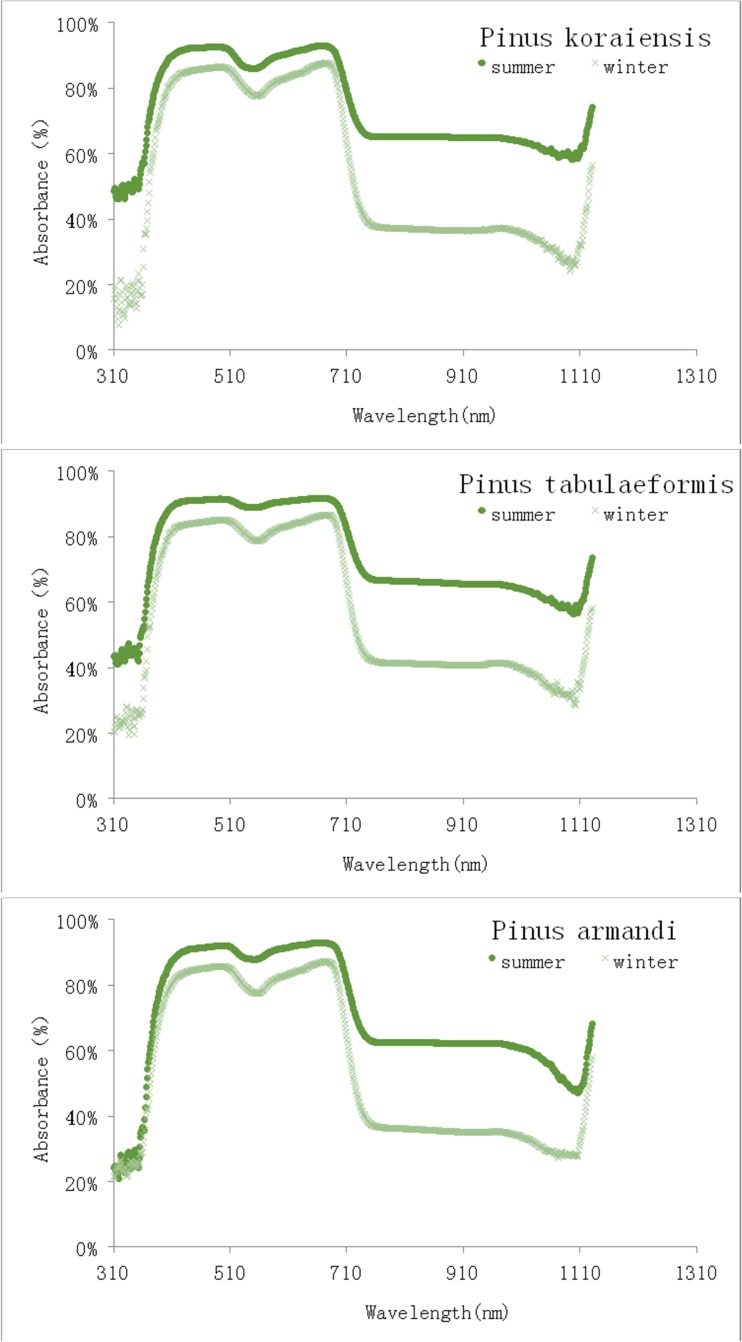
The comparison of absorbance spectra of needles in *Pinus* trees between in cold winter and summer

During very cold winter, photochemical activity is limited by low temperatures, and the needles cannot convert light energy to chemical energy via the photosynthetic electron transfer chain. In this circumstance, all light energy absorbed by leaves is excess excitation energy. To reduce this excess, the needles may decrease light energy absorption. As Fig. 2 shows, in cold winter, the needles absorbed less light energy in the visible range (400–740 nm) than in summer. For instance, the absorbances in summer and winter were, respectively, 92.7 and 86.7 % in *P. koraiensis*, 91.8 and 85.9 % in *P. tabulaeformis*, and 92.9 and 86.3 % in *P. armandi*. These results showed that the leaves in winter still absorbed a large amount of light energy and needed to dissipate a great deal of excess energy. In addition, the decrease in light energy absorption in the winter was not proportional to that in *F*_m_. In all three species, we also observed a significant decrease in absorption in the winter in the infrared light region. The significant decreases in absorption in the UV range were also found in the winter in *P. koraiensis* and *P. tabulaeformis*, but *P. armandi* maintained the same low absorption in the winter and summer. The significance of these changes could not be interpreted.

### Chlorophyll content and chlorophyll *a*/*b* ratio

**Fig. 3:**
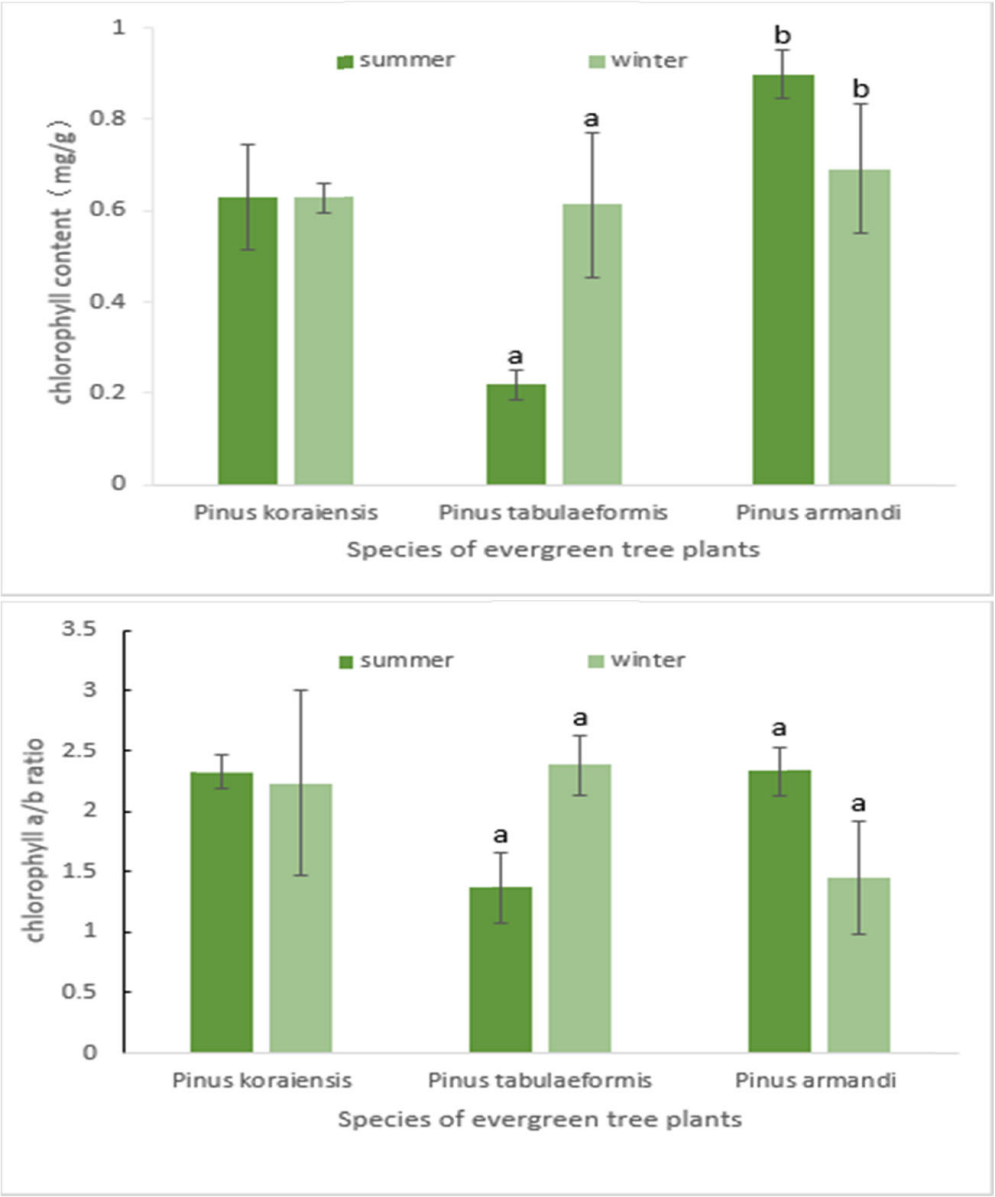
The comparison of chlorophyll content and chlorophyll a/b ratio of needles in *Pinus* trees between in cold winter and summer. The *error bar* is SD. *a*, *b* significance at *P* ≤ 0.01, 0.05, respectively

In this study, the chlorophyll contents and chlorophyll *a*/*b* ratios of the needle leaves from the three pine species did not change consistently between the cold winter and summer (Fig. 3). The analysis of variance showed that the values of both parameters in *P. koraiensis* were not significantly different (*P* ≥ 0.05) in the winter and summer, both values in *P. tabulaeformis* were significantly higher (*P* ≤ 0.05 or ≤0.01) in winter than in summer and both values in *P. armandi* were significantly lower (*P* ≤ 0.01) in winter than in summer. Obviously, changes in the chlorophyll content and chlorophyll *a*/*b* ratio were not correlated with the *F*_m_ decrease in the winter.

### Relative membrane permeability

**Fig. 4:**
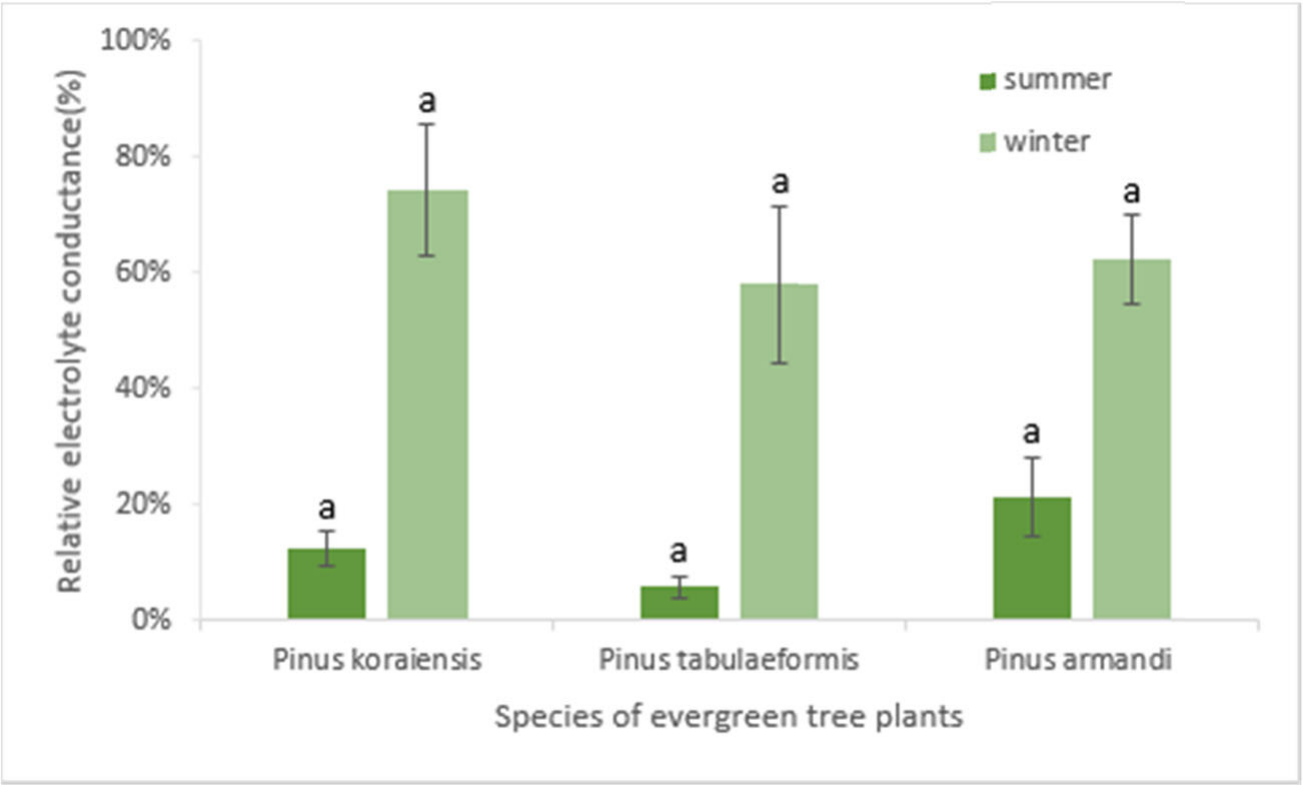
The comparison of relative membrane permeability of needles in *Pinus* trees between in cold winter and summer. The *error bar* is SD. *a* significance at *P* ≤ 0.01

Relative membrane permeability reflects the structural integrity and functional status of the cell membrane. In severe cold, the cell membrane loses its normal double-layer structure and normal function. In winter, the relative membrane permeabilities of the needle leaves from *P. koraiensis*, *P. tabulaeformis*, and *P. armandi* were 74.2, 57.8, and 62.2 %, respectively (Fig. 4). The analysis of variance showed that the relative membrane permeabilities in winter were significantly higher (*P* ≤ 0.01) than in summer. These results showed that the leaves had lost normal physiological activity in winter.

## Discussion and conclusions

Normally, light energy absorbed by leaves is mainly used to reduce carbon dioxide during photosynthesis. When the absorbed energy exceeds the photosynthetic need, the excess energy will dissipate in the form of fluorescence and heat (Allahverdiyeva and Aro 2012; Horton 2012). Although the amount of energy released in fluorescence is a small part of the total energy dissipation, the fluorescence intensity varies with environmental changes (Murata et al. 2007) and with the physiological status of the leaves (Jiang et al. 2006). In this study, the leaf *F*_m_ values in winter leaves were much lower than those in summer in three pine species (Fig. 1 and Table 1). The lower *F*_m_ values may be caused by a decrease in light energy absorption or an increase in photochemical reactions and heat dissipation. Nevertheless, the chlorophyll contents and chlorophyll *a*/*b* ratios varied with species between the winter and summer (Fig. 3) and did not show a coincident change. Obviously, chlorophyll was not the cause of the *F*_m_ decrease in winter. The leaves in winter could not have increased their photochemical reaction rate either, because their biomembranes were non-functional (Fig. 4). In addition, the decrease in light energy absorption in the winter (Fig. 2) was not proportional to the decline in *F*_m_ value (Fig. 1 and Table 1). Therefore, the lower *F*_m_ was mainly attributed to an increase in heat dissipation.

The xanthophyll cycle is an important mechanism for dissipating excess excitation energy as heat (Eskling et al. 1997) during mild winters (Martínez-Ferri et al. 2004). This process involves the enzyme-catalytic interconversion between violaxanthin and zeaxanthin in higher plants (Jahns et al. 2009; Jahns and Holzwarth 2012). During excess light stress, violaxanthin is converted to the intermediate antheraxanthin and then zeaxanthin by violaxanthin de-epoxidase, which requires an acidic pH to bind to the thylakoid membrane (Bratt et al. 1995); the reverse reaction is catalyzed by zeaxanthin epoxidase (Schaller et al. 2012), which functions at pH 7.5 and needs a supply of nicotinamide adenine dinucleotide phosphate, flavin adenine dinucleotide, and O_2_ (Büch et al. 1995). Thus, the operation of the xanthophyll cycle depends on the integrity and normal function of the chloroplast membrane. But in the present study, the relative membrane permeability of the winter needles was 57.8–74.2 %, suggesting the chloroplast membranes had lost their normal structure and physiological functions. Lütz (1996) reported that green leaves of *Eriophorum* that experienced a sudden frost and strong irradiation suffered yellow leaf areas, and high zeaxanthin levels in the affected areas did not prevent the damage. Therefore, we inferred that the heat dissipation of excess energy in the cold winter did not depend on cell function and enzyme activity.

The PSII supercomplex in higher plants contains a reaction center and several light-harvesting pigment complexes (LHCII) (Caffarri et al. 2009). When the green leaves photosynthesize, light energy absorbed by LHCII is transferred to the PSII reaction center, then passes through the cytochrome b_6_-f complex and other transfer intermediates to reach photosystem I (PSI), and is finally used to reduce NADP. While the reaction centers are in close state, the amount of fluorescence released is *F*_m_, which represents the photochemical reaction potential (Strasser and Strasser 1995). Several factors influence the value of *F*_m_, including the state transition and changes in the PSII supercomplex structure (Tikkanen and Aro 2012). During the state transition, LHCII is phosphorylated, moves to the PSI complex (Tikkanen and Aro 2012; Cui et al. 2014), and transfers its energy to PSI reaction center so as to decrease *F*_m_. However, in the low temperatures of the present study, the winter leaves were unable to perform the state transition. Heat release from the PSII supercomplex is an important pathway of energy dissipation, and a relevant protein had been identified from *Chlamydomonas reinhardtii* (Elrad et al. 2002). Therefore, the decrease in leaf *F*_m_ in winter in these three pine species was mainly a result of changes in the PSII complex structure.

In conclusion, the maximum fluorescence of the leaves of the three pine trees decreased substantially in winter. The decrease was not due to a decline in light energy absorption, state transition, or heat dissipation via the xanthophyll cycle. Therefore, the *F*_m_ decrease possibly involved the changes in the PSII supercomplex. This result implies that more attention should be paid to heat dissipation resulting from alterations to the PSII complex architecture during the growing season. Further work is needed to explore the characteristics of the PSII complex architecture in winter and the mechanism of the structural alteration as air temperature declines seasonally.
